# The Use of Digital Tools to Mitigate the COVID-19 Pandemic: Comparative Retrospective Study of Six Countries

**DOI:** 10.2196/24598

**Published:** 2020-12-23

**Authors:** Kylie Zeng, Stephanie N Bernardo, Weldon E Havins

**Affiliations:** 1 College of Osteopathic Medicine Touro University Nevada Henderson, NV United States

**Keywords:** COVID-19, digital tool, policy, proposal, digital health, precaution, spread, contact tracing, public health

## Abstract

**Background:**

Since the COVID-19 outbreak began in Wuhan, China, countries worldwide have been forced to take unprecedented measures to combat it. While some countries are still grappling with the COVID-19 pandemic, others have fared better and have re-established relative normalcy quickly. The rapid transmission rate of the virus has shown a greater need for efficient and technologically modern containment measures. The use of digital tools to facilitate strict containment measures in countries that have fared well against the COVID-19 pandemic has sparked both interest and controversy.

**Objective:**

In this study, we compare the precautions taken against the spread of COVID-19 in the United States, Spain, and Italy, with Taiwan, South Korea, and Singapore, particularly related to the use of digital tools for contact tracing, and propose policies that could be used in the United States for future COVID-19 waves or pandemics.

**Methods:**

COVID-19 death rate data were obtained from the European Center for Disease Prevention and Control (ECDC), accessed through the Our World in Data database, and were evaluated based on population size per 100,000 people from December 31, 2019, to September 6, 2020. All policies and measures enacted were obtained from their respective governmental websites.

**Results:**

We found a strong association between lower death rates per capita and countries that implemented early mask use and strict border control measures that included mandatory quarantine using digital tools. There is a significant difference in the number of deaths per 100,000 when comparing Taiwan, South Korea, and Singapore with the United States, Spain, and Italy.

**Conclusions:**

Based on our research, it is evident that early intervention with the use of digital tools had a strong correlation with the successful containment of COVID-19. Infection rates and subsequent deaths in Italy, Spain, and the United States could have been much lower with early mask use and, more importantly, timely border control measures using modern digital tools. Thus, we propose that the United States execute the following national policies should a public health emergency be declared: (1) immediately establish a National Command responsible for enacting strict mandatory guidelines enforced by federal and state governments, including national mask use; (2) mandate civilian cooperation with health officials in contact tracing and quarantine orders; and (3) require incoming travelers to the United States and those quarantined to download a contact tracing app. We acknowledge the countries we studied differ in their cultures, political systems, and reporting criteria for COVID-19 deaths. Further research may need to be conducted to address these limitations; however, we believe that the proposed policies could protect the American public.

## Introduction

Since the COVID-19 outbreak began, countries across the world have been forced to take unprecedented measures to combat it. By March 13, 2020, the World Health Organization (WHO) officially declared the COVID-19 outbreak as a pandemic [1]. In response, many countries have employed aggressive measures to try to contain the pandemic; the different approaches have yielded varying levels of success. Learning from the harsh experiences of the severe acute respiratory syndrome (SARS) and Middle East respiratory syndrome (MERS) epidemics, Taiwan, Singapore, and South Korea have since enacted legislation and policies in the event of another epidemiological disaster. By employing technology like contact tracing apps and global positioning system (GPS) tracking, these countries have had early success in flattening the curve and keeping death rates low. In contrast, Italy, Spain, and the United States had difficulty managing the spread of the virus despite enforcing national lockdowns and stay-at-home orders. In this study, we compare the precautions taken by various countries against the spread of COVID-19, particularly the use of digital tools in contact tracing, and propose policies that could be used in the United States for future COVID-19 waves or pandemics.

Given the nature of the virus and its rapid transmission, countries around the world have had to rely on nonpharmaceutical measures to mitigate the spread of the virus and reduce the number of new infections. In response, considerable measures including the use of digital contact tracing tools, physical distancing, stay-at-home orders, nationwide lockdowns, and mask requirements have been introduced. Despite these stringent actions, the degree to which these measures were adopted and the subsequent outcomes have varied substantially among countries, with only a few countries having managed to keep the outbreak well controlled.

While stay-at-home orders and nationwide lockdowns assist in “flattening the curve,” they are not economically feasible. Traditionally, contact tracing is performed manually by trained personnel and relies on human memory. However, due to the virus’s rapid transmission and long incubation period, these methods are considered slow, laborious, and are prone to error [2].

Following the 2003 SARS epidemic, Taiwan and Singapore took the necessary legal and structural precautions to prepare for possible future outbreaks [3,4]. More recently, the 2015 MERS outbreak in South Korea resulted in the Korean Government supporting hospitals nationwide in setting up negative pressure rooms [5]. The epidemics these countries have faced in the past 20 years have not only prepared the governments, but also the general public for the current COVID-19 pandemic.

Currently, the use of digital tools to facilitate strict containment measures has sparked both interest and controversy. In Taiwan, mobile phone–based “electronic fencing” uses phone signals to monitor self-quarantine compliance [3]. As for South Korea, in addition to mandated self-isolation protocols, travelers and returning residents are required to install the Self-Quarantine Safety Protection app or Self Diagnosis app, which use mobile GPS tracking. The history of medical facility use and pharmacy visits, credit card transaction logs, and closed circuit television (CCTV) have also been implemented to aid in patient identification [5]. Singapore’s opt-in TraceTogether app uses Bluetooth to exchange time-limited tokens between app users in close proximity, which are then sent to a central server [6]. Although these tools pose privacy concerns, they nonetheless contributed to these countries’ early success in disease management. Countries who had higher death rates, such as the United States, Italy, and Spain, have only recently implemented or considered proposing similar approaches. Although these countries differ in their culture and policies, implementing digital tools in conjunction with other interventions can serve as a useful tool to help countries achieve containment.

## Methods

**Figure 1:**
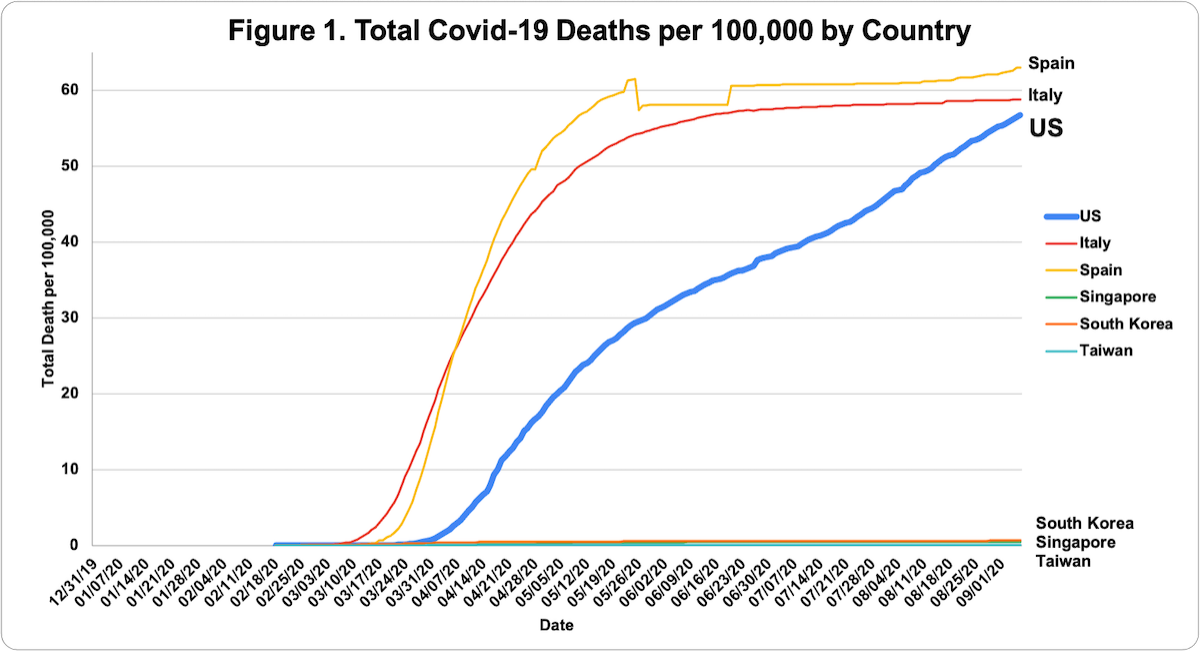
Total COVID-19 deaths per 100,000 people by country from December 31, 2019, to September 6, 2020. Overall, Spain and Italy had the highest total deaths per capita. However, total death rates per capita for both have seem to have plateaued. It is unclear whether the United States (US) is reaching a plateau. Comparatively, Singapore, South Korea, and Taiwan had almost negligible total death rates per capita.

**Figure 2:**
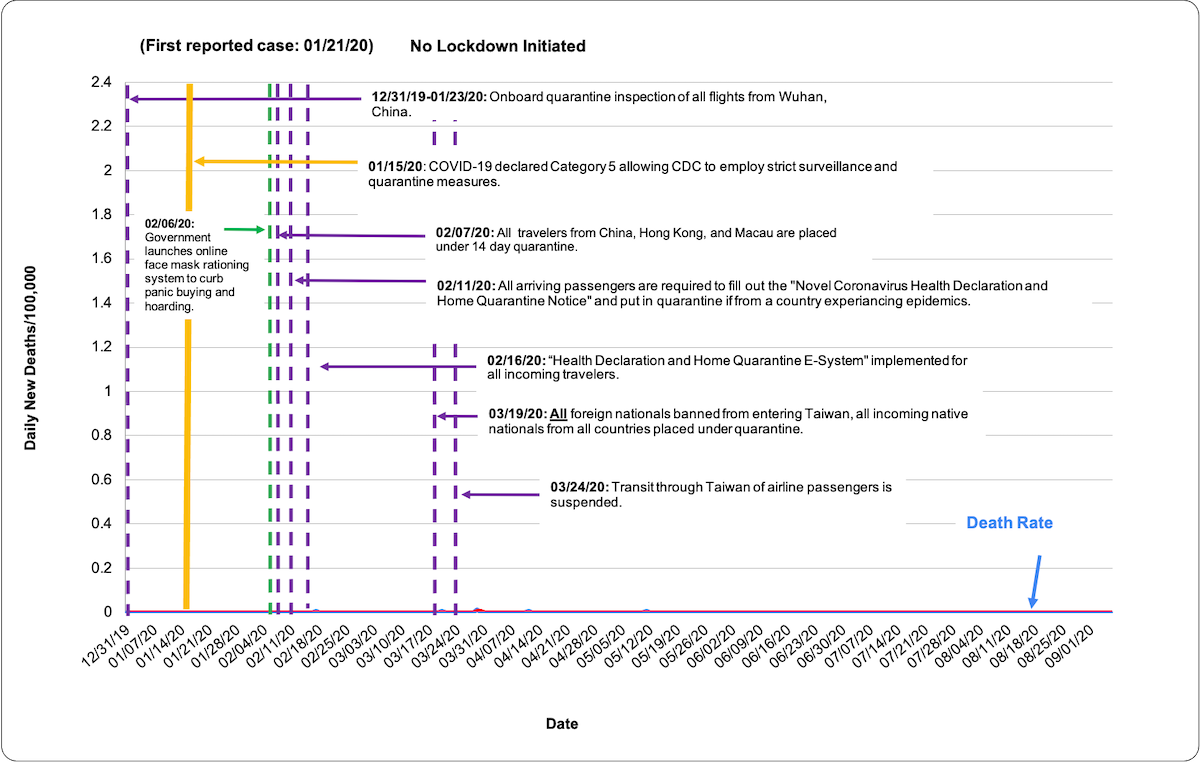
Daily new deaths per 100,000 people for Taiwan from December 31, 2019, to September 6, 2020.

**Figure 3:**
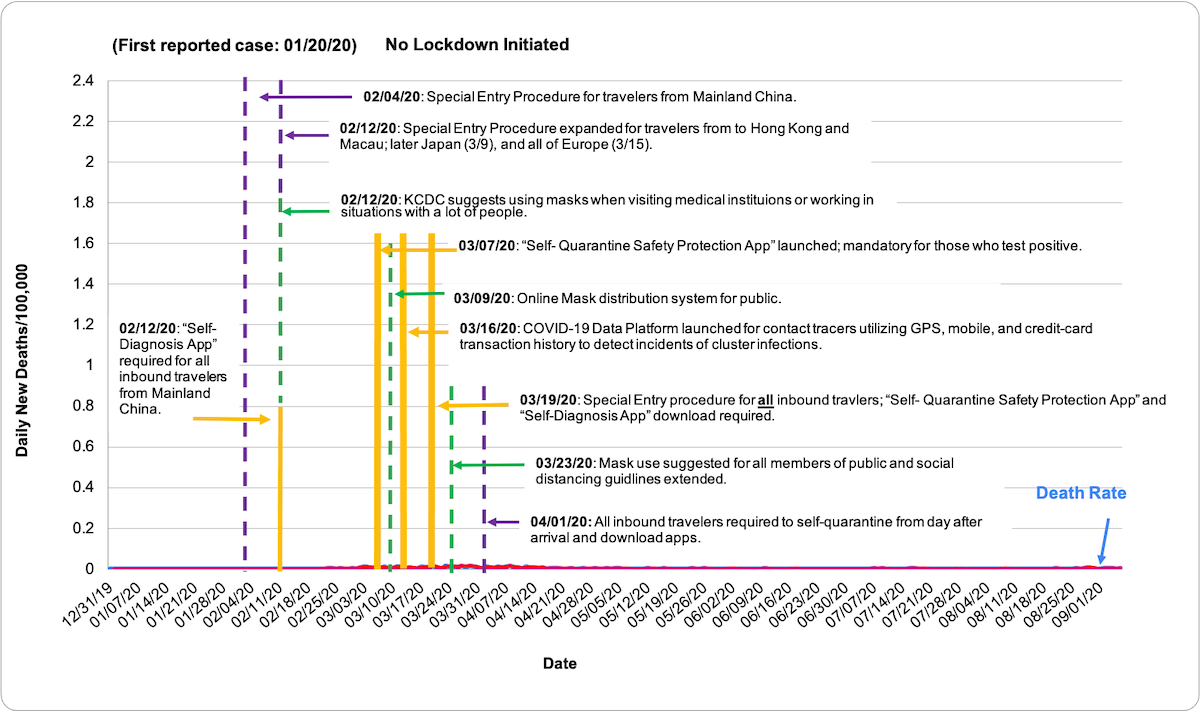
Daily new deaths per 100,000 people for South Korea from December 31, 2019, to September 6, 2020.

**Figure 4:**
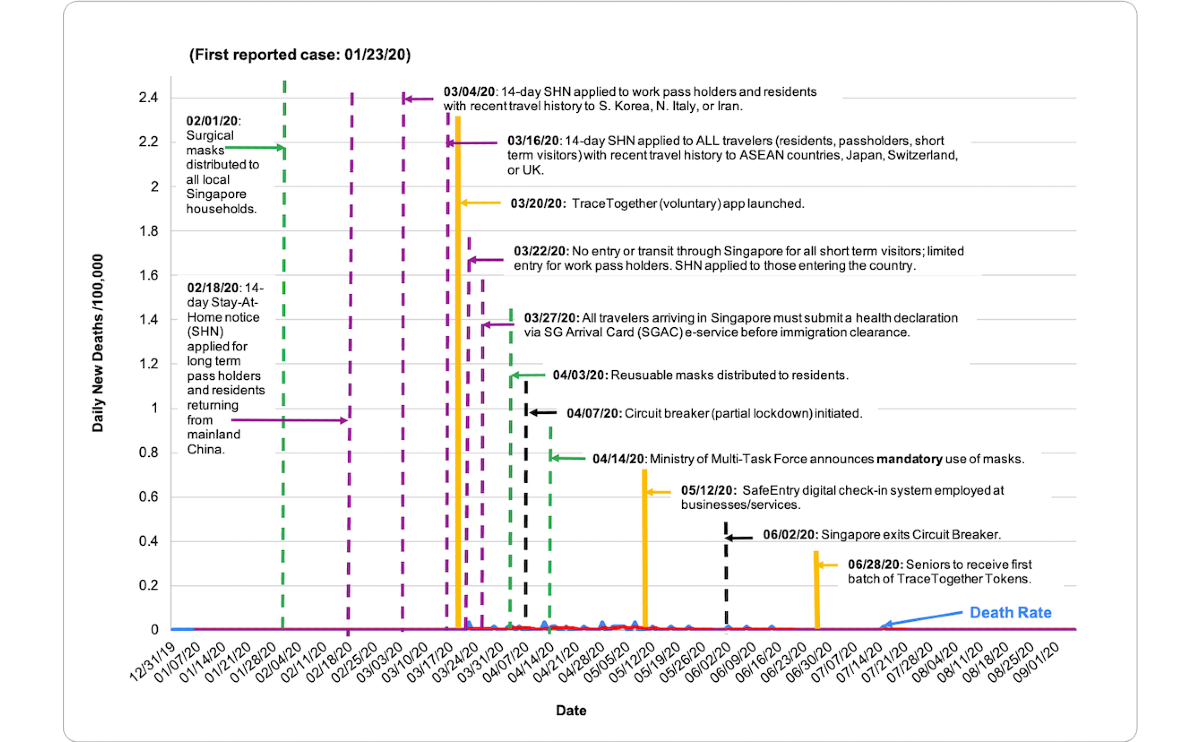
Daily new deaths per 100,000 people for Singapore from December 31, 2019, to September 6, 2020. ASEAN: Association of Southeast Asian Nations; SHN: Stay-Home Notice.

**Figure 5:**
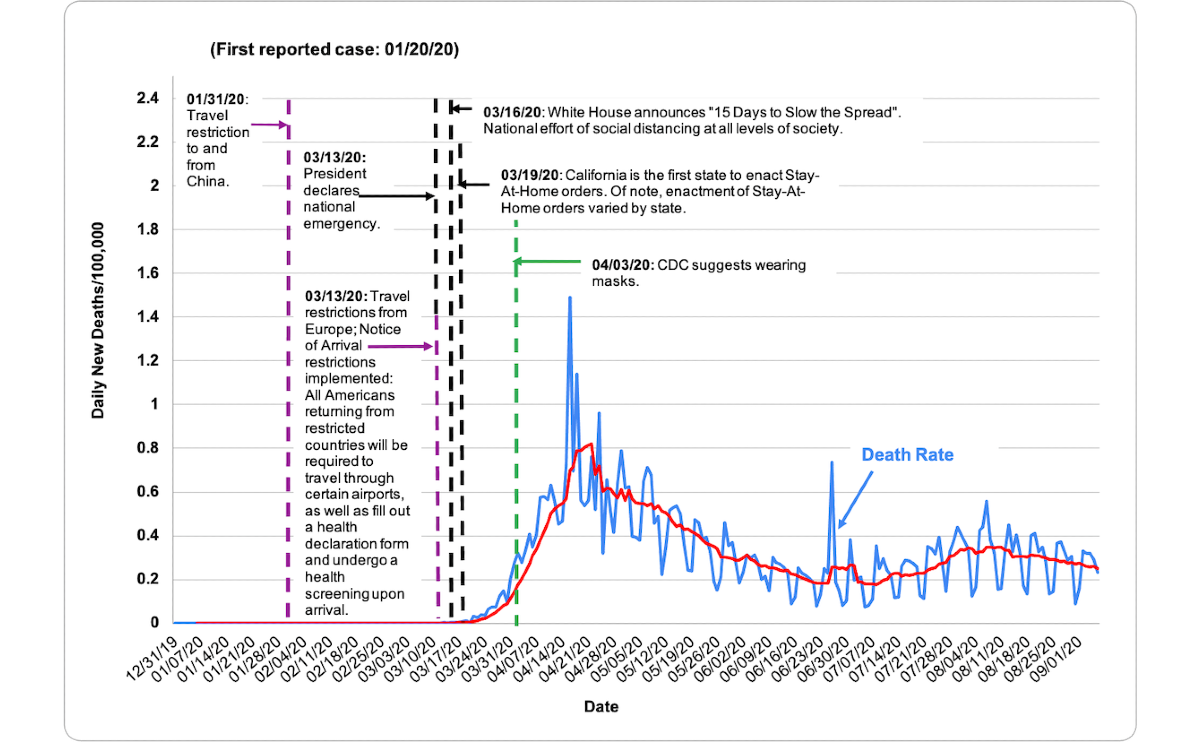
Daily new deaths per 100,000 people for the United States from December 31, 2019, to September 6, 2020.

**Figure 6:**
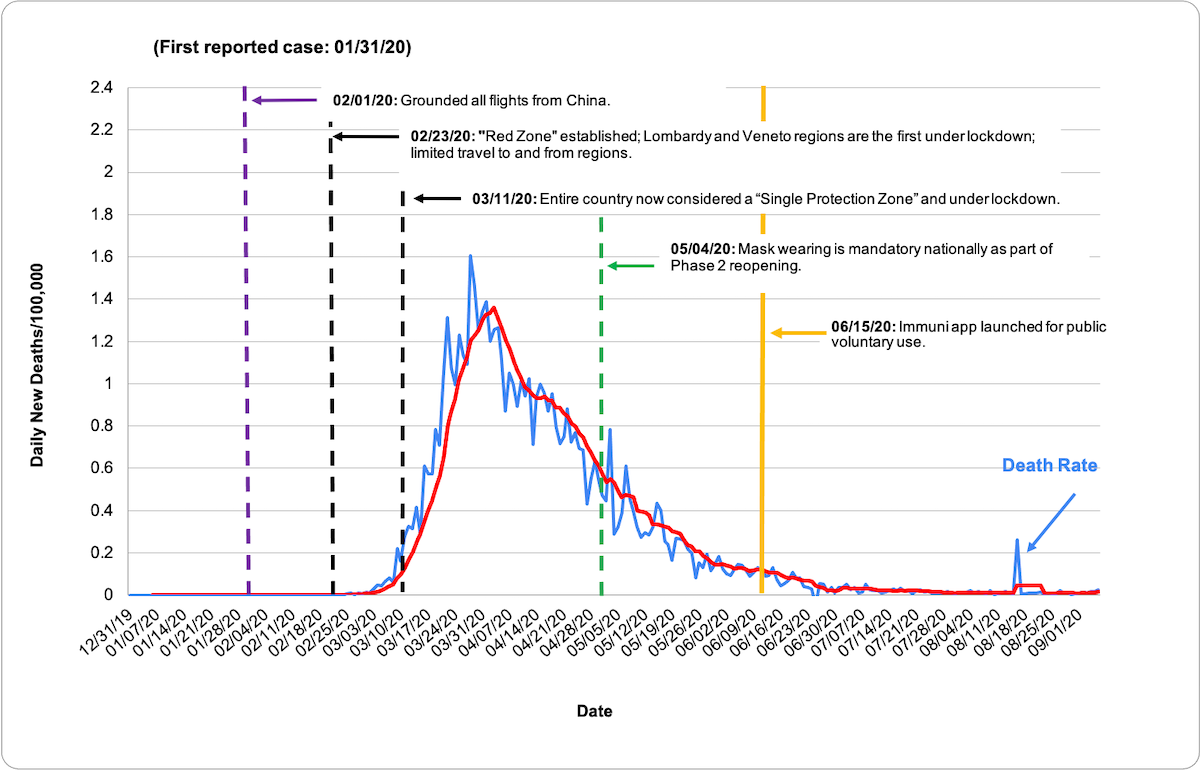
Daily new deaths per 100,000 people for Italy from December 31, 2019, to September 6, 2020.

**Figure 7:**
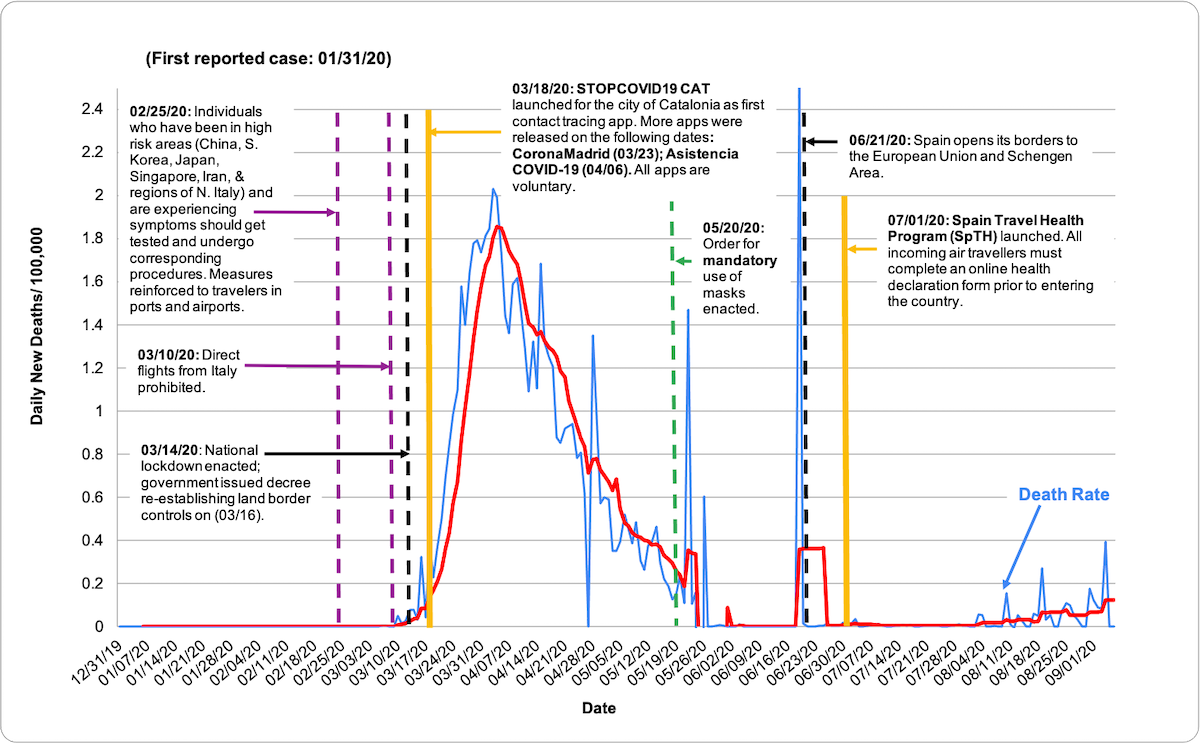
Daily new deaths per 100,000 in Spain from December 31, 2019, to September 6, 2020.

COVID-19 death rate data were obtained from the European Center for Disease Prevention and Control (ECDC), accessed through the Our World in Data database [7]. Death rates were evaluated via descriptive statistics based on population size per 100,000 people from December 31, 2019, to September 6, 2020. Table 1 shows total deaths per 100,000 as of September 6, 2020. Figure 1 shows the total COVID-19 death rate per 100,000 people in each country from December 31, 2019, to September 6, 2020, measured in 7-day intervals. In Figures 2-7, we compared new total daily COVID-19 deaths Taiwan, South Korea, Singapore, the United States, Italy, and Spain. The data is fitted with a moving average trend line. All events labeled on the graphs were obtained from their respective governmental websites. Dates when lockdowns, policies, and contact tracing digital tools were enacted or launched are marked on the graphs. In Figures 2-7, the graph shows the daily new deaths per 100,000 (blue line) for each country, fitted with a 7-day moving average (solid red). Dates digital contact tracing tools were launched (yellow), dates countries were placed on lockdown (black dash), dates masks were suggested/enforced (green dash), and dates border control measures were initiated (purple dash) for each country are marked on the graphs. Policies for Taiwan [3,8], South Korea [5,9-11], Singapore [12-20], the United States [21-24], Italy [25-28], and Spain [29-39] were found on respective governmental websites.

Table 1 compares total COVID-19 deaths per capita between each country. Overall, Spain and Italy have the highest total death per capita, respectively. However, total death rates per capita for both seem to have plateaued. It is unclear whether the United States is reaching a plateau. Comparatively, Singapore, South Korea, and Taiwan have almost negligible total death rates per capita.

**Table 1:** Total COVID-19 deaths per 100,000 people by country as of September 6, 2020.

Country	COVID-19 deaths per 100,000
Spain	62.92
Italy	58.77
United States	56.96
South Korea	0.65
Singapore	0.46
Taiwan	0.03

## Results

As seen in Figure 1, there is a significant difference in the number of deaths per 100,000 when comparing Taiwan, South Korea, and Singapore versus the United States, Spain, and Italy. Most notably, Spain had the highest total number of deaths per 100,000 despite a later initial rise in cases than Italy. Of note, Spain miscounted the number of deaths during the period of May 24-25, 2020, and there have been discrepancies regarding the number of deaths Spain reported to the European CDC for June [7]. Actual values could be higher than reported values given a lack of testing and data lag. Although the United States’ total death per capita is less than that of Italy and Spain, it has not plateaued. Taiwan, Singapore, and South Korea had almost negligible values.

## Discussion

### Principal Findings

While approaches to mitigating the pandemic differ by country, similarities in practices of countries with low death rates have perhaps highlighted what methods are effective. As shown in Figures 2-7, countries with lower death rates per capita implemented strict border control measures that included mandatory quarantine. We found that the use of digital tools and early, centralized action in Taiwan, South Korea, and Singapore were associated with their low COVID-19 death rates and the containment of COVID-19, as seen in Figures 2-4. Additionally, these countries suggested mask use and set up online mask rationing systems during the early stages of the outbreak [3,5,12,13]. Granted, there are cultural differences that may contribute to lower death rates. For example, in Asia, mask-wearing is a social norm, especially after the SARS outbreak [40]. Taiwan, South Korea, and Singapore also implemented measures to provide clear and accurate information to the public, making sure to clarify any misinformation spread among the public swiftly [3-5,14]. The reasons for their success are multifaceted; however, the use of digital tools and early, centralized action are common among Taiwan, South Korea, and Singapore. Countries with higher death rates, such as Spain, Italy, and the United States, operated in a decentralized and antiquated manner. As seen in Figures 5-7, representing the aforementioned countries, it can be seen that policies were enacted just as the death rate started to climb and peak. Although lockdowns in these countries have helped in flattening the curve, they were implemented when it was likely too late to contain the spread of the virus, and high death rates were inevitable.

### Taiwan

With only 7 reported deaths as of September 6, 2020, Taiwan did not initiate a national lockdown [7]. Figure 2 and Table 1 show that Taiwan’s death rate has been low, indicating that their policies enacted well before their first confirmed case allowed them to successfully contain the spread of COVID-19 very early on. Reasons for their success are multifaceted but can in part be attributed to Taiwan’s use of big data analytics and the variety of modern digital tools used to establish mask distribution, strict border control, and mandatory quarantines. Additionally, Taiwan government's took immediate and centralized action. By January 20, 2020, Taiwan’s Central Epidemic Command Center (CECC) was established as a national, centralized agency solely to combat the emerging threat of COVID-19 [3].

The Taiwanese government launched the Name-Based Mask Distribution System on February 3, 2020. This was a national online mask distribution system that allocated masks through local pharmacies and public health centers. This system to allow citizens to purchase masks directly online or through an app called “NHI Express-My Health Bank” [41]. The masks could then be picked up at the closest convenience store [3].

Using technology and lessons learned from the SARS epidemic, Taiwan has standardized fever screenings of arriving airport passengers since 2003 to secure their borders from imported infections. From December 31, 2019, to January 23, 2020, immediately after learning of a possible outbreak in Wuhan, Taiwan began onboard quarantine inspections of all direct flights from Wuhan, China [3]. As more details about COVID-19 began to come to light, Taiwan required all arriving passengers from China, Hong Kong, and Macao (including those transiting through such areas) to fill out a health declaration form and be placed under home quarantine for 14 days as of February 7, 2020. This measure was extended to all incoming passengers on February 11. By February 16, the Taiwanese government launched the “Health Declaration and Home Quarantine E-System” to expedite immigration clearance for all incoming travelers [3]. Travelers with mobile phones can scan a QR code before their flights or upon arrival in Taiwan. A health pass is then sent to their phone via SMS text messaging, which they can then show to immigration for faster clearance. If immigration deems that an individual has to be quarantined, their mobile number will be recorded for GPS surveillance [42]. For those without a Taiwanese mobile number, airport quarantine staff will provide a Taiwanese mobile phone (only at Taoyuan International Airport) or a SIM card [43]. Shortly after the WHO declared the COVID-19 pandemic, Taiwan closed its borders to all foreign nationals on March 19, 2020 [3]. All incoming Taiwanese nationals are required to quarantine for 15 days upon arrival from any country [42]. These border measures may have allowed Taiwan to reduce incoming cases before there was evidence that the virus could spread asymptomatically and before the COVID-19 outbreak escalated into a pandemic.

The use of digital tools in mandatory quarantines has also been attributed to Taiwan’s successful response to the COVID-19 crisis. Using mobile phone positioning technology and big data analytics, Taiwan’s CECC teamed up with 5 major telecom operators to “electronically fence” people placed under quarantine via positioning data of their mobile phones in relation to local cell towers. If the person under quarantine leaves their designated area, local public health officials are notified via SMS text messaging and police can be sent to the quarantine site immediately. In addition, 2 random calls per day are conducted to make sure the person under quarantine has their phone with them [3]. After quarantine is over, geolocation tracking is suspended, and all collected data is deleted as mandated by the Personal Data Protection Act [41]. The application of digital tools to bolster border protocols and mask distribution early in Taiwan seems to have had a significant role in containing COVID-19.

### South Korea

As seen in Table 1, South Korea’s death rate per 100,000 is 0.58, which is low compared to the United States, Spain, and Italy; this was achieved without enforcing a national lockdown [5]. Effectively applying information and communication technology, Korea used GPS tracing and self-quarantine/diagnosis apps to combat the spread of COVID-19. To address the public’s concerns over mask shortages, the South Korean government took measures to develop a distribution system. On March 9, a 5-day mask distribution system was launched to provide the public with a database on mask availability at different retailers [5].

At the border, South Korea implemented special entry procedures for travelers from Mainland China on February 2, 2020, and extended these measures to include Hong Kong and Macau by February 12 [9]. Incoming travelers were required to undergo temperature screenings and download the Self-Diagnosis App, developed by the Korean government to monitor symptoms and provide users with immediate medical advice. After providing identifying information (passport information, nationality, name, address), travelers would have to provide their health condition through the app once per day. Data collected is directly sent to local government public health clinics under the direction of the Korean Center of Disease Control (KCDC). If the traveler reports relevant symptoms for >2 days, local governments can enforce COVID-19 testing. Daily warnings are put in for noncompliance with daily data entering. By the fourth warning, local police are sent to track down the individual. By April 9, the app was downloaded by approximately 170,000 travelers, allowing appropriate action taken for 9000 individuals who developed possible symptoms during their mandatory health monitoring period [5].

On March 7, 2020, the Self-Quarantine Safety Protection App was launched. The app functioned similarly to the Self-Diagnosis app, with the added function of GPS tracking to prevent individuals from violating self-quarantine orders. As of April 1, 2020, all incoming travelers, including Korean nationals, were subject to a 14-day quarantine and required to download one of the apps upon arrival [5]. European and US nationals with short-term visas and all symptomatic inbound travelers were immediately tested at the airport and transferred to quarantine facilities. All others and symptomatic travelers negative for COVID-19 are required to be quarantined for 14 days and download either of the two apps [9]. Overseas roaming data was used to identify and monitor individuals traveling from high-risk countries for the duration of the incubation period [5].

Due to the increasingly heavy workload for contact tracers, the Korean government officially launched the COVID-19 Epidemiological Investigation Support System on March 26, 2020. Governmental agencies collaborated with telecommunication companies, 22 credit card companies, and the Credit Finance Association of Korea to design a streamlined platform that allowed contact tracers to immediately identify transmission routes of an infected individual using GPS location, CCTV, and credit card transactions [5]. This platform significantly expedited the contact tracing process, allowing local contact tracers to easily identify possible cluster outbreaks and act accordingly. Prior to the launch of the COVID-19 Epidemiological Investigation Support System, contact tracers would have to submit location information requests related to patients to the National Police Agencies and various telecommunication companies, which typically took up to 24 hours per case. Handwritten records were kept for each case. The new system expedited this process to 10 minutes per case and allowed for automated tracking records. To protect the privacy of citizens, only KCDC officials and contact tracers in charge have the security clearance to access this data [5].

Additionally, the KCDC releases the movements of de-identified positive cases to the public in an effort to be transparent about possible spread. From this publicly released data, private companies began creating apps that notified users at high risk of exposure and immediately provided directions to the nearest testing sites. Tech companies have also developed apps that use data about masks sold at public retailers provided by the Korean government to make buying them easy for the public [5]. By using digital tools for border containment measures, for streamlined data collection for contact tracers, and to provide transparency to the general public, Korea was able to swiftly employ objective, data-based public health policies to combat COVID-19 both at their borders and within their communities. Thus, the country’s low death rate per 100,000 without enacting a national lockdown may be in part attributed to their use of digital tools in an efficient and responsible manner.

### Singapore

As a city-state consisting of 5.7 million people and serving as one of Southeast Asia’s global travel hubs, Singapore is highly susceptible to the transmission of communicable diseases [14]. It was therefore critical that Singapore act swiftly in their COVID-19 containment efforts. Having experienced the SARS outbreak in 2003, Singapore modified its health care infrastructure to facilitate its response toward future outbreaks [14]. In light of the current pandemic, Singapore’s multifaceted approach toward containment, health care, border control, and community measures has proven to be effective. As of September 6, 2020, Singapore has had a total of 27 reported deaths [7] and 0.46 total deaths per 100,000 people (Table 1).

Prior to its first reported case on January 23, 2020, Singapore had already implemented strict border control measures. As early as January 3, Singapore performed temperature and health screenings for travelers arriving from Wuhan, China. These measures were eventually extended to all ports of entry by January 29 [14]. On January 31, 2020, Singapore introduced a 14-day leave of absence (LOA) to all Singaporean residents and long-term pass holders (LTPHs) returning from Mainland China. Those placed under the LOA were able to leave their place of residence when deemed necessary [44]. However, in a matter of weeks, Singapore’s Multi-Ministry Task Force introduced a stricter “Stay-Home Notice” (SHN), in which Singaporean residents, LTPHs, and short-term visitors entering the country were mandated to remain in their place of residence for 14 days. First applied to travelers returning from Mainland China effective February 18 [15], this mandate was later extended to those with a recent travel history to high-risk countries listed in Figure 4. On March 23, all short-term travelers were banned from entering or transiting through the country [14]. As of March 27, all incoming travelers arriving to Singapore, including residents and LTPHs, are required to submit a health declaration form via the SG-Arrival Card (SGAC) e-service using the web or mobile app before proceeding with immigration clearance. Travelers are also required to comply with the mandated 14-day SHN upon their arrival and are made fully aware of the penalties listed under the Infectious Disease Act should noncompliance ensue [16].

Similar to South Korea and Taiwan, the Singaporean government also heavily suggested public face mask use. On February 1, the government issued 4 surgical masks per household, with priority given to the most vulnerable populations [12]. To address the subsequent rise in cases and shortage of surgical masks, the government later distributed reusable masks to all residents with a registered home address on April 3 [13]. Contrary to its successful counterparts, Singapore mandated mask use in public as of April 14 [17].

To facilitate its contact tracing efforts, Singapore launched the TraceTogether app on March 20 [19]. Using Bluetooth technology, encrypted and anonymized identifiers are exchanged between participating devices in close proximity, which are then securely stored on the users’ devices and a government server that can only be accessed by Singapore’s Ministry of Health (MOH). All data is automatically deleted from users’ devices after it has been stored for 25 days [6]. Should an app user test positive for COVID-19 or become subjected to contact tracing, individuals will be contacted by health authorities and are required by law to share their encounter histories to assist with activity mapping and identifying close contacts [7]. The effectiveness of the app, however, remains questionable. Since its launch, an estimated 20% of the population have downloaded the app [45]. This lack of participation may be attributed to the fact that the app is voluntary. To further support contact tracing capabilities, additional digital tools were introduced. To ease Singapore’s circuit breaker measures and enable safe reopening [46], on May 12, SafeEntry was deployed at entry/exit points of high-traffic areas including public venues, essential services, and businesses. This digital check-in system requires individuals to scan their personalized SafeEntry QR code or have their national identification card scanned at SafeEntry check-in counters [19]. On June 8, the TraceTogether Token was introduced to address digitally excluded populations, such as older adults. Using Bluetooth in the absence of internet or cellular activity, this standalone device can record signals from nearby participating TraceTogether devices [47]. Similarly to the TraceTogether app, all SafeEntry and TraceTogether Token data is encrypted and stored in the government server for a maximum of 25 days, and can only be accessed by health authorities for contact tracing purposes [19,47].

While the TraceTogether app was not as successful as anticipated, the added use of SafeEntry and the TraceTogether Token demonstrates that Singapore remains heavily reliant on technology for contact tracing purposes. The use of these digital tools in addition to strict border measures and health precautions nonetheless demonstrates the significant role technology can have in epidemiological control.

### United States

Although the United States has a lower total number of COVID-19 deaths per capita (56.96) when compared to Spain and Italy (Table 1), the total number of cases and deaths in the United States as of September 6, 2020, stand at 6,245,866 and 188,538, respectively. Additionally, while Spain and Italy seem to have reached a plateau in their total number of deaths per 100,000, as seen in Figure 1, the United States’ numbers continue to increase. Although the United States appears to show a downward trend from mid-April to June, as of July, the death rate appears to be rising again (Figure 5).

The outcomes of the COVID-19 pandemic in the United States have been less than ideal, in part due to the country’s delayed actions and uncoordinated national response. The first COVID-19 case in the United States was reported on January 20, 2020. However, it was not until March 13 that a national emergency was declared; the “15 Days to Slow The Spread” national effort was endorsed 3 days thereafter [21]. Despite declaring a national emergency, a nationwide lockdown was not initiated. Similar to Italy and Spain, the United States responded to the pandemic in a “patchwork” manner. State governments and localities were primarily responsible for enforcing their public health measures, while the federal government provided minimal support. As a result, policies regarding lockdowns, border control, contact tracing, and mask use have varied by state [22,23,48,49].

Since its first reported case in January, the United States applied travel bans to China (January 31), Iran (February 29), and Europe (March 11) [21]. On March 13, the Department of Homeland Security issued a Notice of Arrival Restriction. Under this order, American citizens, legal permanent residents, and immediate family members returning to the United States from high-risk COVID-19 areas can only travel through certain airports and are required to undergo an enhanced entry screening that includes completing a health declaration form upon arrival. Symptomatic travelers are referred to the Centers for Disease Control and Prevention (CDC) for further evaluation. Asymptomatic travelers are instructed to self-quarantine and monitor their health in accordance with CDC guidelines, and are contacted by health officials to ensure compliance with these measures [24]. However, policies regarding domestic travel have varied by state. Although some states require incoming travelers to provide documentation, go through a health screening, complete COVID-19 testing, or quarantine, the majority of states do not have mandatory travel-related restrictions [50].

At the start of the pandemic, there had been limited evidence regarding asymptomatic transmission. Additionally, there were concerns about mask shortages for health care workers. As a result, US public health authorities did not initially recommend mask use in public. However, as the number of cases rose and new evidence regarding virus transmission was released, on April 3, the CDC issued guidelines recommending the use of cloth face masks in public. Although this was not required nationally, between April and May, only 15 states had issued mandates requiring the public use of face masks. Although these mandates have been issued, mandatory mask use and compliance has become a social and political issue in the United States [51]. However, with the surge in cases occurring since reopening the economy, the number of states requiring masks to be worn statewide or at the local level has increased to 48 [51].

With regard to contact tracing and quarantine orders, while the CDC provides the guidance and resources pertaining to these measures, state and local health departments are responsible for their practices [50]. Prior to the pandemic, the United States used conventional methods of contact tracing, which heavily rely on the use of public health personnel to conduct extensive follow-up with infected individuals and close contacts, as well as manually log this information. However, the current pandemic has demonstrated that these methods are not feasible [2]. The CDC estimates that the United States will need about 100,000 contact tracers to contain the next wave. Currently, many local health departments have only a limited number of trained personnel to pursue this task [52]. Given these circumstances, other methods to increase contact tracing capacity have been considered, including hiring more government employees, recruiting volunteers, and using digital tools [53]. Although some states have developed contact tracing apps or considered using them, many states have been reluctant to use such technology given privacy concerns and a lack of public adoption. As a result, many states have opted to rely solely traditional practices [54]. We believe that efforts to use this digital tool on a national level would be more effective.

Despite actions taken at the state and local level, the United States’ delayed and decentralized response may have been a contributing factor to its high death rates. Had the United States enacted early universal policies using digital tools for border control, mask distribution, and contact tracing, these actions could have significantly reduced community transmission. As the number of cases and deaths continue to rise, it is all the more important that federal, state, and local governments work with each other and the public to combat the virus.

### Italy

By July 30, 2020, Italy’s death rate per 100,000 was 58.10 (Table 1). As cases and deaths began to rise [7], the Italian government initiated lockdowns incrementally via territories. Starting on February 23, 2020, 11 municipalities were designated as the “Red Zone.” Italy gradually extended regions for lockdown, but was ultimately forced to designate the entire country as a “protection zone” and implement a national lockdown on March 11 [25]. During these lockdowns, citizens were asked to avoid any unnecessary excursions outside the home and were required to have a self-declaration form on hand when in public [55]. Checkpoints were put in place by the police to ensure that the public was in compliance with these measures [56]. Additionally, as of March 17, the government required citizens returning from abroad to self-isolate for 14 days [57]. Although Italy’s lockdown did eventually help flatten the curve, these measures were implemented at a time where such drastic measures were likely necessary to mitigate what would already inevitably be a high death rate. Figure 6 shows that the death rate peaked around March 31, 2020, three weeks after the national lockdown was implemented. It is likely that the spread of the virus in Italy at this time was beyond the threshold for containment measures.

Italy’s contact tracing and quarantine surveillance method relied heavily on traditional methods. Local public health operators are to call infected individuals to obtain information about the person’s movement, areas of stay in the past 14 days, and possible close contacts. After getting in touch with potentially infected individuals, contact tracers are required to convey basic COVID-19 information and supervise adherence to quarantine measures by calling these individuals on a daily basis. Furthermore, contact tracers are required to explain the methods and purposes of home isolation if it is decided that an individual will have to be put under quarantine [25]. Given the transmission method and incubation of COVID-19, such traditional contact tracing methods may be slow and inefficient, especially as the workload for contact tracers surges. Although Italy did impose a mandatory quarantine for those who tested positive, they implemented harsher penalties for violation of quarantine after their lockdown was enacted, around the time the death rate reached its peak, on March 26, 2020 [7].

As the country started to enter their Phase 2 of reopening, the Italian government took various precautions to ensure safe reopening. As of May 4, 2020, wearing masks in public was made mandatory nationally [55]. To enforce greater security at the border, travelers entering Italy after July 9, 2020, from any country other than European Union member states, the United Kingdom, Andorra, the Principality of Monaco, the Republic of San Marino, and Vatican City State are required to undergo a 14-day quarantine period and carry a self-declaration form with them in case they are stopped by law enforcement officers. Individuals put under mandatory self-isolation have to do so immediately upon entering the country [58]. Additionally, on June 15, the Italian Ministry of Health launched Immuni, a voluntary Bluetooth-based contact tracing app that the government developed to contain possible future waves [58]. The app uses a randomly generated code that is emitted continuously via Bluetooth, similar in function to Singapore’s TraceTogether app. All data collected from this app are deleted by the Ministry of Health when no longer relevant, and no later than December 31, 2020 [26]. To protect the privacy rights of its citizens, the Italian Government issued a legal decree on April 30, 2020, establishing that all user data would be anonymized or pseudonymized, geolocation information collection would be strictly prohibited, and that data collected by the Ministry of Health would only be used for epidemiological purposes. This required the government to provide clear and transparent information about the data collected [59]. Despite a difficult first wave, Italy has updated its border control and health policies in preparation for a potential second wave.

### Spain

Among the countries studied, Spain has had the highest total death rate per capita at 62.92 per 100,000 people (Table 1). Of note, there have been discrepancies in how Spain has reported their data. This is depicted in Figure 1 and Figure 7. During the period of May 24-25 and early June, the Spanish government addressed miscounted deaths, delays in reporting data, low testing capacity, and changes to its COVID-19 reporting criteria [60,61]. Therefore, it is possible that the total number of deaths per capita may be higher given these factors. 

On January 31, 2020, Spain confirmed its first COVID-19 case. At that time, Spain had also initiated the repatriation process of its citizens from Wuhan, China. All repatriated citizens were required to undergo an extensive health screening upon their return and were transferred by bus to Gomen Ulla Hospital for further evaluation and quarantine despite being asymptomatic [62]. On February 25, stricter measures were enforced at airports and all ports of entry into the country, particularly for travelers coming from high-risk COVID-19 areas [29]. Given the concerns about the growing number of cases, lockdown measures ensued. Comparable to Italy and the United States, Spain initially approached lockdowns regionally, beginning with the region of Haro on March 7 and four Catalan municipalities on March 12 [63,64]. However, by March 14, the government issued a royal decree declaring a state of alarm and a nationwide lockdown was enacted. Spanish citizens were required to limit their mobility unless deemed essential. Nonessential businesses shut down and face-to-face activities were suspended. Additionally, land border controls were re-established, which allowed only Spanish citizens, residents, cross-border workers, and those who could prove causes of force majeure via documentation to enter the country [30]. To ensure compliance with lockdown measures and social distancing practices, areas were also heavily policed, with some areas using drones [30,65]. Despite implementing these strict measures, it seems that the spread of the virus outpaced Spain’s ability to contain it. As seen in Figure 7, Spain reached its peak number of deaths around April 3 [7]. Had Spain implemented these practices early on, the number of lives lost could have been significantly reduced.

Similarly to Italy and the United States, Spain resorted to traditional forms of contact tracing, which consist of local health authorities extensively interviewing infected individuals and their close contacts, and closely following up with them via phone calls during their 14-day quarantine period [66]. In an effort to prevent the unnecessary use of emergency services and overwhelming hospital capacity, several autonomous communities released their own regionally based COVID-19 apps [31-33]. These voluntary apps allow users to self-assess their symptoms and then provide users with the best health precautions to take. If a user meets certain health criteria, they may be contacted by local health authorities. Additionally, users have the option to provide their geolocation data, which is used to provide them with local resources, and for COVID-19 heat-mapping purposes. Geofencing is not performed. All personal and health data, protected by Spain’s Protection of Personal Data law, are managed by the Ministry of Health and will only be kept for the remainder of the current health crisis [34-36]. Currently, not much is known about the effectiveness of these apps. Therefore, it is difficult to assess whether or not these apps have contributed to Spain’s downward trend in its number of deaths per capita.

Since introducing its “plan for transition toward a new normal” on April 28, Spain has introduced new policies to ensure safe reopening [67]. As of May 15, all incoming travelers are required to quarantine for 14 days in their place of residence [68]. On May 19, public mask use was made mandatory [69]. The government also introduced the Spain Travel Health program on July 1, which requires incoming travelers to fill out an online health declaration form 48 hours prior to their trip. Travelers must then present their personalized QR code upon their arrival [37]. By implementing some practices similar to those of Taiwan, South Korea, and Singapore, these actions could positively contribute to Spain’s response in future waves or pandemics.

### Conclusion

While most of the world’s developed countries have been able to endure the first wave of the COVID-19 pandemic, the United States is still struggling to combat this virus effectively. As of September 6, 2020, the United States recorded a total of 188,538 deaths due to COVID-19 [7]. It is imperative that appropriate measures be taken in the case of future waves or pandemics. The low death rates of certain countries have suggested that the use of digital tools, along with strong adherence to social distancing measures, may be an associated measure in effectively flattening the curve early. From our research, we believe that there is a strong association between the use of these digital tools and low death rates in Taiwan, Singapore, and South Korea. We believe that this association is substantial enough to suggest the use of such digital tools in the United States. These methods have raised privacy concerns, but the countries that used them have legally established provisions and guidelines that protect both the privacy and health of their citizens. Provisions are established under Taiwan’s Personal Data Protection Act, South Korea’s COVID-19 Epidemiological Investigation Support System guidelines, and through the terms of use for SafeEntry and TraceTogether [5,19,41,47]. Italy, in an attempt to avoid the great loss of life they endured in their first wave, has implemented a national data security law pertaining to contact tracing apps [26].

Granted, there are countries, like New Zealand, who have low COVID-19 death rates without using digital tools [70]. However, we believe that despite the low death rates achieved by such countries, this would be less plausible in the United States. Given the population size of the United States, the enormity of the data that would need to be evaluated to establish containment measures would likely benefit from using digital tools as Taiwan, South Korea, and Singapore have. Moreover, New Zealand has also opted to use digital tools to mitigate a second wave with its NZ COVID Tracer app [71]. The United States recently introduced the use of digital tools in their vaccination efforts. On October 14, 2020, the CDC announced their plans to use V-SAFE, a smartphone-based system aimed at monitoring the side effects of the COVID-19 vaccine [72].

Although some of the methods that countries like Taiwan, Singapore, and South Korea have employed may seem invasive to Americans, the loss of American lives has been substantial. Based on our findings, we propose that the United States adopts the following national policies to be temporarily enforced based on the findings of public health officials, with a supermajority of Congress and the President agreeing to declare a public health state of emergency. The circumstances needed to justify these contract tracing methods would be narrowly tailored to a compelling government interest where a great number of American lives would be at risk. They are as follows:

1. National command: During early signs of a possible epidemic or pandemic, a national task force should be formed with the head of the CDC and NIH at the helm. Mask use will be mandated nationally early. Strict mandatory guidelines on how to address the pandemic will be outlined and provided to states. Working with the private sector, this task force will oversee the increased production of critical medical supplies. National stockpiles should be established and maintained to last up to a year.
2. Contact tracing and quarantine: Individuals and close contacts are required to cooperate with public health officials. They will be required to download a contact tracing app on their phones that will be used to track and monitor their quarantine and symptoms. The app will use Bluetooth and GPS technology to ensure proper quarantine protocols are being followed. Data collected will only be used and accessed by the CDC and will be deleted after quarantine
  .
  Those without a smartphone will be monitored by local health officials via phone. Laws regarding the protection of digital data will be promulgated.
3. Border control: During the early stages of an epidemiological crisis, require all incoming travelers to download the contact tracing app and fill out a health questionnaire daily. If symptoms arise, they are required to immediately notify the local health official assigned to them and be tested. Information on who to notify will be provided to the traveler via the app.

We acknowledge the countries we studied differ in their cultures, political systems, and reporting criteria for COVID-19 deaths. Further research may need to be conducted to address these limitations.

## Abbreviations

CCTV: closed circuit television
CDC: Center for Disease Control
CECC: Taiwan’s Central Epidemic Command Center
ECDC: European Center for Disease Prevention and Control
GPS: global positioning system
KCDC: Korean Center of Disease Control
LOA: leave of absence
LTPH: Singaporean long-term pass holders
MERS: Middle East respiratory syndrome
SARS: severe acute respiratory syndrome
SGAC: Singapore-Arrival Card
SHN: Stay-Home Notice
WHO: World Health Organization
